# Recognition of Intrabiliary Hepatic Metastases From Colorectal Adenocarcinoma

**DOI:** 10.1155/2000/17619

**Published:** 2000-08

**Authors:** Stephen P. Povoski, David S. Klimstra, Karen T. Brown, Lawrence H. Schwartz, Robert C. Kurtz, William R. Jarnagin, Yuman Fong, Leslie H. Blumgart

**Affiliations:** 1 Department of Surgery Memorial Sloan – Kettering Cancer Center New York New York USA; 2 Department of Pathology Memorial Sloan – Kettering Cancer Center New York New York USA; 3 Department of Radiology Memorial Sloan – Kettering Cancer Center New York New York USA; 4 Department of Medicine Memorial Sloan – Kettering Cancer Center New York New York USA; 5 Section of Surgical Oncology Department of Surgery West Virginia University Morgantown West Virginia USA; 6 Department of Surgery Memorial Sloan – Kettering Cancer Center 1275 York Avenue New York New York 10021 USA

## Abstract

Intrinsic involvement of bile ducts, by metastatic colorectal adenocarcinoma growing from within or invading the lumen of bile ducts, is not a well recognized pattern of tumor growth. Clinical, radiographic, operative, and histopathologic aspects of 15 patients with intrabiliary colorectal metastases were described. Fourteen patients were explored for possible hepatic resection. Two had jaundice, two radiographic evidence of an intrabiliary filling defect, 10 intraoperative evidence of intrabiliary tumor, and six microscopic evidence of intrabiliariy tumor. Eleven patients underwent hepatic resection. Five of the resected patients developed hepatic recurrence. Four patients were explored for possible repeat resection. One had jaundice, one radiographic evidence of an intrabiliary filling defect, all had intraoperative evidence of intrabiliary tumor, and three microscopic evidence of intrabiliary tumor. Three patients underwent repeat hepatic resection. All patients with preoperative jaundice and radiographic evidence of an intrabiliary filling defect were unresectable. Overall, actuarial five-year survival is 33% for those patients resected *versus* 0% for those not resected. Intraoperative recognition of intrabiliary tumor at exploration for hepatic resection was more common than clinical, radiographic, or histopathologic recognition. More diligent examination of resected liver tissue by the surgeon and pathologist may increase identification of bile duct involvement and aid in achieving adequate tumor clearance.


*HPB Surgery*, 2000, Vol. 11, pp. 383-391
Reprints available directly from the publisher
Photocopying permitted by license only

© 2000 OPA (Overseas Publishers Association) N.V.
Published by license under
the Harwood Academic Publishers imprint,
part of The Gordon and Breach Publishing Group.
Printed in Malaysia.

### Recognition of Intrabiliary Hepatic Metastases from Colorectal Adenocarcinoma

STEPHEN P. POVOSKI[a,*], DAVID S. KLIMSTRA[b], KAREN T. BROWN[c], LAWRENCE H. SCHWARTZ[c], ROBERT C. KURTZ[d], WILLIAM R. JARNAGIN[a], YUMAN FONG[a] and LESLIE H. BLUMGART[a,†]

[a]*Departments of Surgery,* [b]*Pathology,* [c]*Radiology,* [d]*Medicine, Memorial Sloan-Kettering Cancer Center, New York, New York, USA*

*(Received 16 May 1999; In final form 25 October 1999)*

**Intrinsic involvement of bile ducts, by metastatic colorectal adenocarcinoma growing from within or invading the lumen of bile ducts, is not a well recognized pattern of tumor growth. Clinical, radiographic, operative, and histopathologic aspects of 15 patients with intrabiliary colorectal metastases were described. Fourteen patients were explored for possible hepatic resection. Two had jaundice, two radiographic evidence of an intrabiliary filling defect, 10 intraoperative evidence of intrabiliary tumor, and six microscopic evidence of intrabiliary tumor. Eleven patients underwent hepatic resection. Five of the resected patients developed hepatic recurrence. Four patients were explored for possible repeat resection. One had jaundice, one radiographic evidence of an intrabiliary filling defect, all had intraoperative evidence of intrabiliary tumor, and three microscopic evidence of intrabiliary tumor. Three patients underwent repeat hepatic resection. All patients with preoperative jaundice and radiographic evidence of an intrabiliary filling defect were unresectable. Overall, actuarial five-year survival is 33% for those patients resected *versus* 0% for those not resected. Intraoperative recognition of intrabiliary tumor at exploration for hepatic resection was more common than clinical, radiographic, or histopathologic recognition. More diligent examination of resected liver tissue by the surgeon and pathologist may increase identification of bile duct involvement and aid in achieving adequate tumor clearance.**

*Keywords*: Colorectal cancer; Metastases; Biliary; Hepatic

### INTRODUCTION

There are approximately 131,000 new cases of colorectal cancer per year in the United States [1]. Approximately 50% of these patients will develop recurrence within five years of treatment of the primary colorectal cancer, with the liver representing the site of recurrence in 40% to 80% of cases [2-5]. The vast majority of hepatic colorectal metastases represent parenchymal lesions. Diffuse parenchymal disease, which is usually a late finding in patients with extensive tumor involvement of the liver, is the usual cause of jaundice [6]. Much less frequently,

*Present address: Section of Surgical Oncology, Department of Surgery, West Virginia University, Morgantown, West Virginia, USA.
†Address for correspondence: Department of Surgery, Memorial Sloan-Kettering Cancer Center, 1275 York Avenue, New York, New York 10021. Tel.: 212-639-5526, Fax: 212-794-5852, e-mail: blumgarl@mskcc.org jaundice can be caused solely by involvement of the bile ducts and, when present, is predominantly due to extrahepatic extrinsic compression [7–12]. Such extrahepatic obstruction generally involves metastases to lymph nodes situated behind the duodenum, along the common bile duct, or in the porta hepatis. Intrinsic involvement of the bile ducts, either by metastatic colorectal adenocarcinoma growing primarily from within intrahepatic or extrahepatic bile ducts or invading into the lumen of such ducts, is not a well recognized pattern of spread of hepatic colorectal metastases and has rarely been reported in the literature [7, 13–16]. In the present paper, we report on the clinical, radiographic, operative, and histopathologic findings of intrabiliary hepatic colorectal metastases.

### PATIENTS AND METHODS

Between January, 1991 and January, 1998, 15 cases of intrabiliary hepatic colorectal metastases were identified by the Hepatobiliary Surgical Service at Memorial Sloan–Kettering Cancer Center from approximately 900 patients evaluated with hepatic colorectal metastases. During this same time period, a total of 430 patients were operatively explored for hepatic colorectal metastases, of which 313 patients underwent hepatic resection. The cases of intrabiliary hepatic colorectal metastases were discovered either during the preoperative radiographic evaluation, at operative exploration for attempted hepatic resection (by gross recognition of intrabiliary tumor at the time of transection of the bile duct structures and/or transection of the liver parenchyma), at pathologic examination, or at autopsy. Clinical, radiographic, operative, and histopathologic findings were examined and contrasted for patients at the time of presentation of initial hepatic metastases and recurrent hepatic metastases. Preoperative radiographic studies, consisting of computed tomography (CT, $n = 15$), CT arterial portography (CTAP, $n = 8$), ultrasound (US, $n = 11$), magnetic resonance cholangiopancreatography (MRCP, $n = 5$), endoscopic retrograde cholangiopancreatography (ERCP, $n = 4$), and percutaneous transhepatic cholangiography (PTHC, $n = 3$), were identified. The original interpreting radiologists' reports were reviewed for identification of intrabiliary filling defects and bile duct dilatation. Reanalysis of radiographic studies at the time of this analysis was not possible since many studies were performed at outside hospitals and were not currently available. The original interpreting pathologists' reports were reviewed for evidence of intrabiliary tumor and then histopathologic slides were reexamined by a single pathologist (D.S.K.) at the time of this analysis. Hepatic resections were classified according to the Couinaud classification [17,18]. Kaplan–Meier survival analysis was used to estimate survival time.

### RESULTS

### Demographics

Fifteen patients with intrabiliary hepatic colorectal metastases were identified between January, 1991 and January, 1998. These patients consisted of 10 men and 5 women. They had a median age of 70 years (range 45–80).

### Initial Hepatic Colorectal Metastases

The median time interval from resection of the primary colorectal adenocarcinoma to clinical presentation of hepatic metastases was 31 months (range 0–80). Clinical presentation of hepatic metastases included the finding of synchronous hepatic metastases in three patients, jaundice in three patients, and an elevated carcinoembryonic antigen (CEA) in 9 patients. Abnormal laboratory values included an elevated CEA in 14 patients, an elevated serum alkaline phosphatase in nine patients, and an elevated serum total bilirubin in three patients.

Fourteen of 15 patients were explored for possible hepatic resection. Eleven patients underwent hepatic resection, two underwent surgical biliary enteric bypass, and one had placement of an hepatic artery pump. One patient, presenting with jaundice, was radiographically determined unresectable secondary to multiple bilobar hepatic lesions and was found on both CT and PTHC to have a filling defect in the distal common bile duct consistent with intrabiliary tumor. Bile duct cytology showed malignant cells consistent with adenocarcinoma. This patient was not explored and a percutaneous transhepatic stent was placed.

The clinical, radiographic, operative and histopathologic evidence of intrabiliary hepatic colorectal metastases was compared for those 14 patients explored (Tab. I). Two had jaundice preoperatively, two preoperative radiographic evidence of an intrabiliary filling defect (Figs. 1 A–C), five preoperative radiographic evidence of biliary dilatation (Figs. 1B, C), 10 gross evidence of intrabiliary tumor intraoperatively, and six microscopic evidence of intrabiliary tumor on initial histopathologic examination. When histopathology was reexamined at the time of this analysis, microscopic evidence of intrabiliary tumor was noted in 10 of 14 patients explored. One patient explored but not resected and who did not have gross evidence of intrabiliary tumor intraoperatively was, 19 months later at autopsy, found to have gross and microscopic evidence of intrabiliary tumor.

Of 11 patients undergoing hepatic resection (Tab. I), none had jaundice preoperatively, none preoperative radiographic evidence of an intrabiliary filling defect, two preoperative radiographic evidence of biliary dilatation, eight gross evidence of intrabiliary tumor intraoperatively, and four microscopic evidence of intrabiliary tumor on initial histopathologic examination. When histopathology was reexamined at the

TABLE I Comparison of the clinical, radiographic, operative, and histopathologic evidence of intrabiliary tumor in 15 patients with initial hepatic colorectal metastases

| Case | Clinical findings | Radiographic findings | Operative findings | Operation | Histopathologic findings (initial exam/reexamination) |
|---|---|---|---|---|---|
| 1 | jaundice | IBT, BDD | IBT[a] | biliary enteric bypass | IBT/IBT |
| 2 | none | none | IBT | right hepatectomy | none/IBT |
| 3 | none | BDD | IBT | left hepatectomy | IBT/IBT |
| 4 | none | BDD | IBT | right lobectomy | none/none |
| 5 | none | BDD | none | hepatic artery pump | none/none[b] |
| 6 | none | none | none | right hepatectomy | none/IBT |
| 7 | none | none | IBT | right hepatectomy | IBT/IBT |
| 8 | none | none | IBT | segmentectomy (segment 4,5) | none/IBT |
| 9 | jaundice | IBT, BDD[c] | N/A | N/A | IBT/IBT[d] |
| 10 | none | none | IBT | left hepatectomy | IBT/IBT |
| 11 | jaundice | IBT, BDD | IBT[a] | biliary enteric bypass | IBT/IBT |
| 12 | none | none | none | right lobectomy | none/none |
| 13 | none | none | none | wedge resection | none/none |
| 14 | none | none | IBT | right lobectomy | none/IBT |
| 15 | none | none | IBT | right hepatectomy | IBT/IBT |

BDD = bile duct dilation; IBT = intrabiliary tumor; N/A = not applicable.
[a]This patient had intrabiliary tumor found intraoperately within the bile ducts, did not undergo hepatic resection, and had a biliary enteric bypass.
[b]Intrabiliary tumor was found in the bile ducts at autopsy approximately 19 months after placement of hepatic artery pump.
[c]This patient had hepatic disease which was radiographically determined unresectable, did not come to exploration, and had a percutaneous transhepatic stent placed.
[d]Cytology was positive for adenocarcinoma at percutaneous transhepatic cholangiography.

A

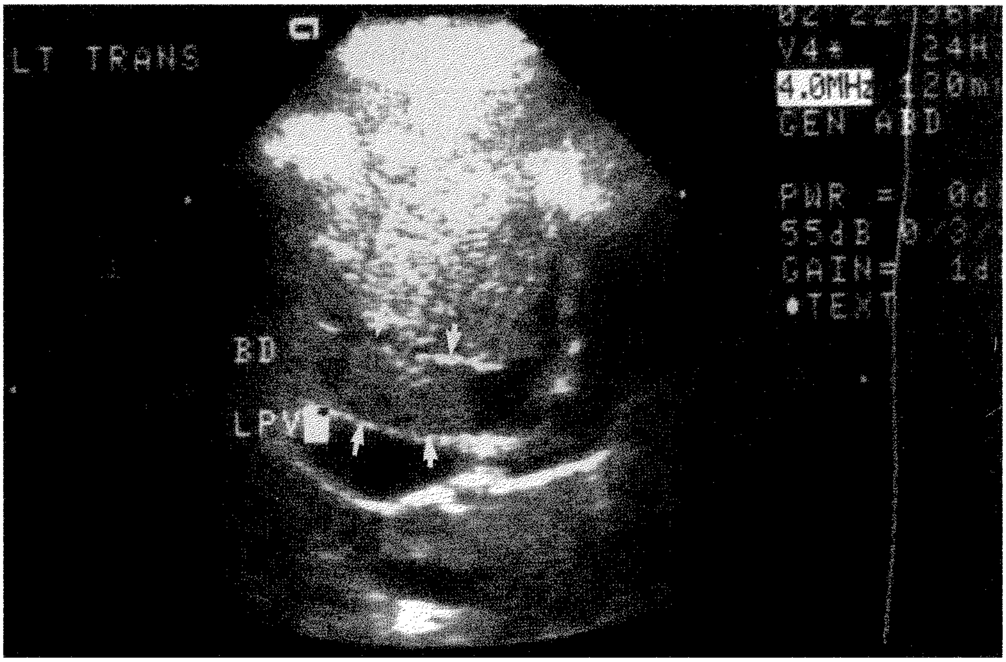

B

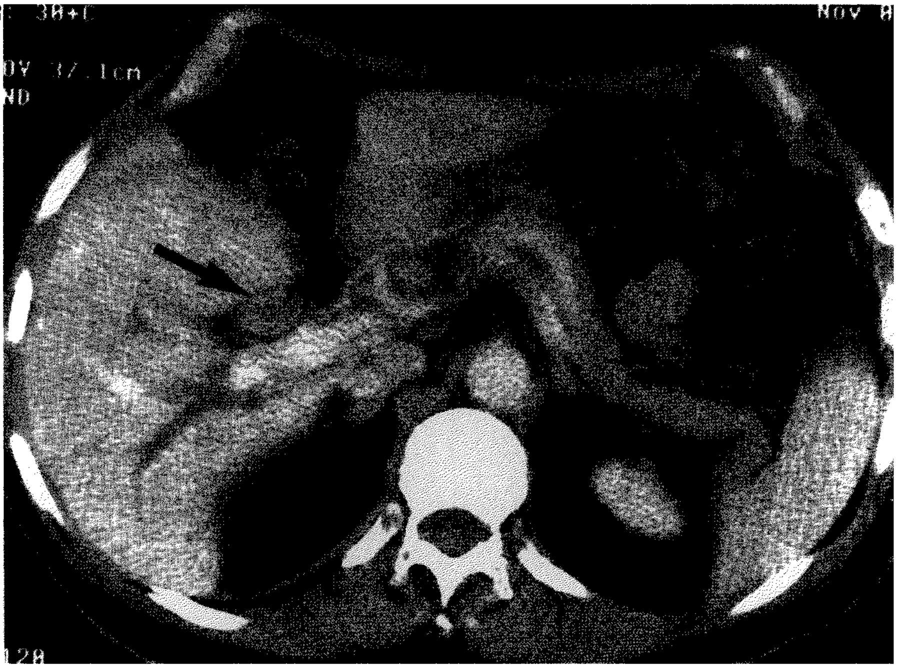

FIGURES 1A–C Transverse ultrasound image (Fig. 1A) demonstrating a filling defect within the common duct (outlined with white arrows). Axial computed tomography (CT) image (Fig. 1B) demonstrating intrahepatic biliary dilatation and a filling defect within the common duct (black arrow). Also note portal lymphadenopathy. Coronal (Fig. 1C) source images from a magnetic resonance cholangiopancreatography (MRCP) demonstrating biliary dilatation (solid white arrow) and abrupt cutoff of the common duct (open white arrow) with an intermediate signal intensity filling defect (white asterisk) within the common duct.

C

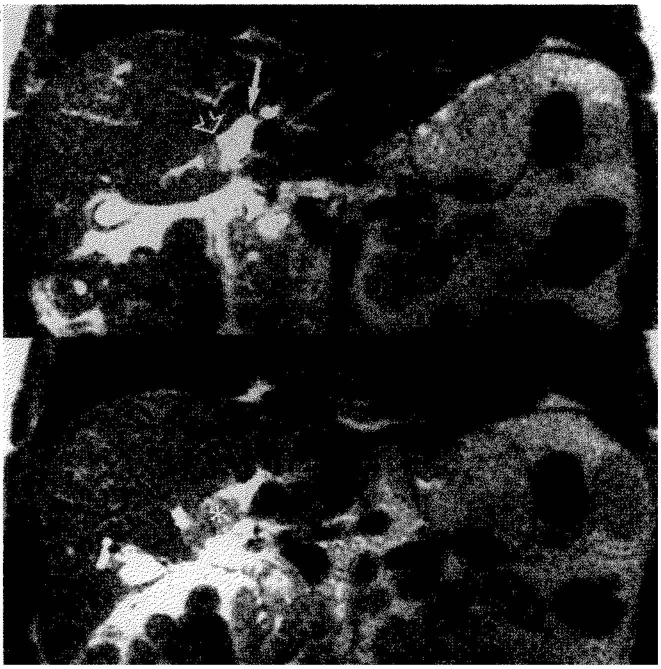

FIGURES 1A–C (Continued)

time of this analysis, microscopic evidence of intrabiliary tumor was noted in eight of 11 patients undergoing hepatic resection.

### Recurrent Hepatic Colorectal Metastases

To date, five of 11 patients undergoing hepatic resection have developed recurrent hepatic metastases. The median time interval from resection of hepatic metastases to clinical presentation of recurrent disease was 23 months (range 22–35). Clinical presentation of recurrence included jaundice in two patients and an elevated CEA in three patients. Abnormal laboratory values included an elevated CEA in four patients, an elevated serum alkaline phosphatase in four patients, and an elevated serum total bilirubin in two patients.

Four of five patients were explored for possible repeat hepatic resection. Three patients underwent repeat hepatic resection and one patient underwent surgical biliary enteric bypass. One patient, presenting with jaundice, was radiographically determined at PTHC to have high grade obstruction at the confluence of the right anterior and posterior sectoral hepatic ducts which was consistent with intrabiliary tumor. Bile duct cytology showed malignant cells consistent with adenocarcinoma. This patient was not explored and a percutaneous transhepatic stent was placed.

The clinical, radiographic, operative and histopathologic evidence of intrabiliary hepatic colorectal metastases was compared for those 4 patients explored (Tab. II). One had jaundice preoperatively, one preoperative radiographic evidence of an intrabiliary filling defect and biliary dilatation, all had gross evidence of intrabiliary tumor intraoperatively, and three microscopic evidence of intrabiliary tumor on initial histopathologic examination. When histopathology was reexamined at the time of this analysis, microscopic evidence of intrabiliary tumor was noted in all four patients explored.

Of three patients undergoing repeat hepatic resection (Tab. II), none had jaundice preopera-

TABLE II Comparison of the clinical, radiographic, operative, and histopathologic evidence of intrabiliary tumor in five patients with recurrent hepatic colorectal metastases

| Case | Clinical findings | Radiographic findings | Operative findings | Operation | Histopathologic findings (initial exam/reexamination) |
|---|---|---|---|---|---|
| 2 | jaundice | IBT, BDD | IBT[a] | biliary enteric bypass | IBT/IBT |
| 3 | jaundice | IBT, BDD[b] | N/A | N/A | IBT/IBT[c] |
| 6 | none | none | IBT | left lobectomy | IBT/IBT |
| 12 | none | none | IBT | segmentectomy (segment 4) | none/IBT |
| 13 | none | none | IBT | right lobectomy | IBT/IBT |

BDD = bile duct dilatation; IBT = intrabiliary tumor; N/A = not applicable.
[a]This patient had intrabiliary tumor found intraoperately within the bile ducts, did not undergo hepatic resection, and had a biliary enteric bypass.
[b]This patient had hepatic disease which was radiographically determined unresectable, did not come to exploration, and had a percutaneous transhepatic stent placed.
[c]Cytology was positive for adenocarcinoma at percutaneous transhepatic cholangiography.

tively, none preoperative radiographic evidence of an intrabiliary filling defect or biliary dilatation, all had gross evidence of intrabiliary tumor intraoperatively, and two microscopic evidence of intrabiliary tumor on initial histopathologic examination. When histopathology was reexamined at the time of this analysis, microscopic evidence of intrabiliary tumor was noted in all three patients undergoing repeat hepatic resection.

### Follow-up

Median follow-up for all patients is 19 months (0–59 months). For those 11 patients undergoing hepatic resection, actuarial five-year survival is 33% with a median actuarial survival of 52 months (range 0–59). For those four patients with unresectable hepatic metastases, actuarial five-year survival is 0% with a median actuarial survival of 14 months (range 7–19).

### DISCUSSION

Intrinsic involvement of the bile ducts either by metastatic colorectal adenocarcinoma growing primarily from within intrahepatic or extrahepatic bile ducts or invading into the lumen of such ducts is not a well recognized pattern of growth of hepatic colorectal metastases and has rarely been reported in the literature. Two cases of intrabiliary hepatic colorectal metastases were first described in the literature by Herbut and Watson in 1946 [7]. In 1982, Gray *et al.*, described a case of metastatic colorectal adenocarcinoma to the liver in which tumor extended into the lumen of the common bile duct [13]. In 1984, Roslyn *et al.*, described three cases of metastatic colorectal adenocarcinoma to the liver in which intrabiliary tumor debris caused obstruction at a site distant from the main hepatic tumor mass and caused intermittent, episodic biliary obstruction with a clinical picture similar to choledocholithiasis [14]. Recently, Riopel *et al.*, have elegantly outlined the histopathologic features of eight cases of intrabiliary growth of hepatic colorectal metastases [16]. They emphasized that the intrabiliary growth of hepatic colorectal metastases along intact basement membranes of bile ducts may make it difficult to distinguish metastatic colorectal lesions from primary bile duct neoplasms.

Recently, Yamamoto *et al.*, specifically examined the mode of extension of hepatic colorectal metastases in 40 consecutive patients undergoing hepatic resection at the National Cancer Center Hospital in Tokyo, Japan [15]. They reported that 20% of patients ($n = 8$) had gross bile duct invasion, commonly with papillary growth in the ductal lumen extending from intraparenchymal metastatic lesions.

Microscopically, tumor invasion to the bile ducts was observed in 40% of patients ($n = 16$). These results would suggest that the phenomenon of intrabiliary growth and extension of hepatic colorectal metastases is much more common than previously thought. There are two potential reasons why this phenomenon may frequently go unrecognized. First, since not all patients with hepatic colorectal metastases undergo extensive biliary imaging, hepatic resection, or biliary enteric bypass/stenting, many cases of intrabiliary involvement may go undetected. Second, once a patient comes to hepatic resection, if the pattern of intrabiliary involvement and spread of hepatic metastases is not specifically looked for on gross and microscopic evaluation of resected liver tissue, it may go unrecognized.

In the present study, we identified 15 cases of intrabiliary hepatic colorectal metastases. Intraoperative recognition of intrabiliary tumors at the time of exploration for possible hepatic resection was more common than was clinical, radiographic, or histopathologic recognition. However, reexamination of the histopathology at the time of this analysis did confirm all but one case of intrabiliary tumor identified intraoperatively. This emphasizes the importance of the surgeon, at the time of hepatic resection, carefully examining the resected liver tissue for gross tumor involvement and extension along or within the bile ducts. Similarly, this emphasizes the importance of the pathologist microscopically examining the resected liver tissue specifically for bile duct involvement. It is our belief that recognition of this phenomenon supports a policy of wide anatomical resection for hepatic colorectal metastases and should warrant further resection of involved hepatic parenchyma and bile ducts, if intrabiliary tumor is identified intraoperatively at the resection margin. Indeed, this was applicable in our series for eight patients resected with initial hepatic metastases and for three patients resected with recurrent hepatic metastases.

Preoperative jaundice, as shown in Tables I and II, was present in three of 15 patients with initial hepatic metastases and two of five patients with recurrent hepatic metastases. All patients with preoperative jaundice had obvious radiographic evidence of an intrabiliary filling defect and none of these patients were amendable to hepatic resection. Therefore, preoperative jaundice, although present in only in minority of patients with intrabiliary tumor, correlates with preoperative radiographic recognition of an intrabiliary filling defect as well as the intraoperative determination of unresectability.

To date, there is no evidence as to the effect of intrabiliary tumor on the survival of patients after hepatic resection for hepatic colorectal metastases. For the 11 patients in our series undergoing hepatic resection, actuarial five-year survival is 33%, which is comparable to most large series evaluating survival after hepatic resection for hepatic colorectal metastases [19–25], including our own experience [26]. A central question is whether intrabiliary tumor is responsible for unrecognized tumor involvement of hepatic margins at hepatic resection, even though the liver parenchymal margin itself is negative. In the paper by Yamamoto *et al.*, they emphasized the importance of recognizing tumor spread along bile ducts and portal vein radicals for obtaining complete tumor clearance [15]. However, they did not evaluate the effect of finding these factors on postresectional survival. Likewise, our small cohort of patient does not allow us to address this issue. This question would be best addressed in the future by a prospective clinicopathologic study in which all resected liver specimens are specifically examined for ductal and vascular involvement.

While intrabiliary hepatic colorectal metastases are not a well recognized pattern of growth of metastatic hepatic colorectal lesions, recent evidence suggests that this phenomenon is more common than previously thought. We believe that more diligent intraoperative gross examination of resected liver tissue by the surgeon may increase identification of bile duct involvement. In turn, this may lead to more appropriate utilization of intraoperative histopathologic examination by frozen section to verify such findings. Taken together, this may aid in achieving adequate tumor clearance in such cases. Nevertheless, the impact of finding intrabiliary hepatic colorectal metastases on survival of patients undergoing hepatic resection remains uncertain.

### *Reference*

[1] Landis, S. H., Murray, T., Bolden, S. and Wingo, P. A., Cancer statistics, 1998. *CA. Cancer J. Clin.*, **48**, 6–29.

[2] Pestana, C., Reitemeier, R. J., Moertel, C. G., Judd, E. S. and Dockerty, M. B. (1964). The natural history of carcinoma of the colon and rectum. *Am. J. Surg.*, **108**, 826–829.

[3] Bross, I. D. J., Viadana, E. and Pickren, J. (1975). Do generalized metastases occur directly from the primary? *J. Chron. Dis.*, **28**, 149–159.

[4] Welch, J. P. and Donaldson, G. A. (1979). The clinical correlation of an autopsy study of recurrent colorectal cancer. *Ann. Surg.*, **189**, 496–502.

[5] Clarke, D. N., Jones, P. F. and Needham, C. D. (1980). Outcome in colorectal carcinoma: seven-year study of a population. *Br. Med. J.*, **280**, 431–435.

[6] Jaffe, B. M., Donegan, W. L., Watson, F. and Spratt, J. S. (1968). Factors influencing survival in patients with untreated hepatic metastases. *S.G.O.*, **127**, 1–11.

[7] Herbut, P. A. and Watson, J. S. (1946). Metastatic cancer of the extrahepatic bile ducts producing jaundice. *Am. J. Clin. Pathol.*, **16**, 365–372.

[8] Nagler, J. and Rochwarger, A. M. (1977). Metastatic colon carcinoma simulating primary bile duct carcinoma *via* endoscopic cholangiography. *Gastrointest. Radiol.*, **2**, 75– 76.

[9] Sung, M. W., Bruckner, H. W., Szabo, S. and Mitty, H. A. (1988). Extrahepatic obstructive jaundice due to colorectal cancer. *Am. J. Gastroenterology*, **83**, 267–270.

[10] Warshaw, A. L. and Welch, J. P. (1978). Extrahepatic biliary obstruction by metastatic colon carcinoma. *Ann. Surg.*, **188**, 593–597.

[11] Thomas, J. H., Pierce, G. E., Karlin, C., Hermreck, A. S. and MacArthur, R. I. (1981). Extrahepatic biliary obstruction secondary to metastatic cancer. *Am. J. Surg.*, **142**, 770–773.

[12] Meyer, J. E., Messer, R. J. and Patel, V. C. (1978). Diagnosis and treatment of obstructive jaundice secondary to liver metastases. *Cancer*, **41**, 773–775.

[13] Gray, R. R., Mackenzie, R. L. and Alan, K. P. (1982). Cholangiographic demonstration of carcinoma of the colon metastatic to the lumen of the common bile duct. *Gastrointest. Radiol.*, **7**, 71–72.

[14] Roslyn, J. J., Kuchenbecker, S., Longmire, W. P. and Tompkins, R. K. (1984). Floating tumor debris. A cause of intermittent biliary obstruction. *Arch. Surg.*, **119**, 1312–1315.

[15] Yamamoto, J., Sugihara, K., Kosuge, T., Takayama, T., Shimada, K., Yamasaki, S., Sakamoto, M. and Hirohashi, S. (1995). Pathologic support for limited hepatectomy in the treatment of metastases from colorectal cancer. *Ann. Surg.*, **221**, 74–78.

[16] Riopel, M. A., Klimstra, D. S., Godellas, C. V., Blumgart, L. H. and Westra, W. H. (1997). Intrabiliary growth of metastatic colonic adenocarcinoma: a pattern of intrahepatic spread easily confused with primary neoplasia of the biliary tract. *Am. J. Surg. Path.*, **21**, 1030–1036.

[17] Couinaud, C. (1957). *Le Foie. Etudes Anatomiques et Chirurgicales*. Paris: Masson.

[18] Blumgart, L. H. (1994). Liver resection – liver and biliary tumours. In: *Surgery of the Liver and Biliary Tract*, edited by Blumgart, L. H., 2nd edn., pp. 1495–1537. Edinburgh: Churchill Livingstone.

[19] Fortner, J. G., Silva, J. S., Golbey, R. B., Cox, E. B. and Maclean, B. J. (1984). Multivariate analysis of a personal series of 247 consecutive patients with liver metastases from colorectal cancer. I. Treatment by hepatic resection. *Ann. Surg.*, **199**, 306–316.

[20] Butler, J., Attiyeh, F. F. and Daly, J. M. (1986). Hepatic resection for metastases of the colon and rectum. *S.G.O.*, **162**, 109–113.

[21] Bradpiece, H. A., Benjamin, I. S., Halevy, A. and Blumgart, L. H. (1987). Major hepatic resection for colorectal liver metastases. *Br. J. Surg.*, **74**, 324–326.

[22] Hughes, K. S., Rosenstein, R. B., Songhorabodi, S., Adson, M. A., Ilstrup, D. M., Fortner, J. G., Maclean, B. H., Foster, J. H. and Daly, J. M. (1988). Resection of the liver for colorectal carcinoma metastases: a multi-institutional study of long-term survivors. *Dis. Colon Rectum*, **31**, 1–4.

[23] Hughes, K. S., Simon, R., Songhorabodi, S., Adson, D. M., Ilstrup, D. M., Fortner, J. G., Maclean, B. J., Foster, J. H. and Daly, J. M. (1988). Resection of the liver for colorectal carcinoma metastases: a multi-institutional study of indications for resection. *Surgery*, **103**, 278–288.

[24] Steele, G. and Ravikumar, T. S. (1989). Resection of hepatic metastases from colorectal cancer. Biological perspectives. *Ann. Surg.*, **210**, 127–138.

[25] Steele, G., Bleday, R., Mayer, R. J., Lindblad, A., Petrelli, N. and Weaver, D. (1991). A prospective evaluation of hepatic resection for colorectal carcinoma metastases to the liver: gastrointestinal tumor study group protocol 6584. *J. Clin. Oncol.*, **9**, 1105–1112.

[26] Fong, Y., Cohen, A. M., Fortner, J. G., Enker, W. E., Turnbull, A. D., Coit, D. G., Marrero, A. M., Prasad, L. H., Blumgart, L. H. and Brennan, M. F. (1997). Liver resection for colorectal metastases. *J. Clin. Oncol.*, **15**, 938–946.

### COMMENTARY

The authors have drawn attention to the involvement of the bile ducts by metastatic colorectal adenocarcinoma, by reporting 15 cases from their large experience of 430 patients explored. The cases resected with intrabiliary tumour represented approximately 4% of their series of resected patients and is therefore, not insignificant. They pointed out that with this report and other recent evidence, the occurrence of bile duct involvement is probably more common than previously thought.

The question arises regarding the clinical significance of bile duct involvement. Although this is a small cohort of their experience, the survival would appear to be commensurate with patients who do not have such involvement and also with reported series of resection for colorectal liver metastases.

In the jaundiced patients, all had preoperative radiographic evidence of intrabiliary tumour and none were amenable to resection. Except for the non-explored patient who had bilateral unresectable disease, the reason for non-resectability was not stated. It is uncertain whether non-resectability was related to intrabiliary tumour or other reasons. However, it was very clear that preoperative jaundice due to intrabiliary tumour as shown on radiology, correlated with non-resectability.

The intrabiliary tumour was recognised at "the time of transection of the bile duct structures or transection of the liver parenchyma". This is interpreted as transecting through tumour at this site, followed by further wider resection of the duct/parenchyma to gain clearance. Scheele has reported that re-resection to gain a clear margin subsequent to identification of a macroscopic positive margin fails to give the patient a survival advantage over patients who had non-curative resection-median survival of 18 months [1]. Although this does not refer specifically to bile duct involvement, it is difficult to perceive the difference. However, there is a difference with the present series reporting a median actuarial survival of 52 months.

Obviously, it would be an advantage to avoid transecting tumour but how can this be achieved? Evidence of bile duct dilatation on preoperative imaging or raised biliary enzymes should alert the surgeon to the possibility of intrabiliary tumour. This report has highlighted biliary tract involvement by metastatic colorectal adenocarcinoma and should prompt surgeons to identify such by intraoperative ultrasound and thereby negotiate a wider resection of the area to avoid transecting the tumour.

### *Reference*

[1] Hughes, K., Scheele, J. and Sugarbaker, P. H. (1989). Surgery for colorectal cancer metastatic to the liver. *Surg. Clin. Nth. Am.*, **69**, 344.

Prof R. W. Strong
Director of Surgery
Princess Alexandra Hospital
Ipswich Road
Wooloongabba
QLD 4102 Australia

